# Phylogeography of *Petrolisthes armatus*, an invasive species with low dispersal ability

**DOI:** 10.1038/s41598-017-03410-8

**Published:** 2017-06-13

**Authors:** Alexandra Hiller, Harilaos A. Lessios

**Affiliations:** Smithsonian Tropical Research Institute, Box 0843-03092, Balboa, Panama

## Abstract

Theoretically, species with high population structure are likely to expand their range, because marginal populations are free to adapt to local conditions; however, meta-analyses have found a negative relation between structure and invasiveness. The crab *Petrolisthes armatus* has a wide native range, which has expanded in the last three decades. We sequenced 1718 bp of mitochondrial DNA from native and recently established populations to determine the population structure of the former and the origin of the latter. There was phylogenetic separation between Atlantic and eastern Pacific populations, and between east and west Atlantic ones. Haplotypes on the coast of Florida and newly established populations in Georgia and South Carolina belong to a different clade from those from Yucatán to Brazil, though a few haplotypes are shared. In the Pacific, populations from Colombia and Ecuador are highly divergent from those from Panamá and the Sea of Cortez. In general, populations were separated hundreds to million years ago with little subsequent gene flow. High genetic diversity in the newly established populations shows that they were founded by many individuals. Range expansion appears to have been limited by low dispersal rather than lack of ability of marginal populations to adapt to extreme conditions.

## Introduction

The population-genetic constitution of marine invasive species in their native range is increasingly being studied in efforts to determine the source of invasions into new areas (reviews in refs 1–5). Less attention has been focused on the insights that such studies can provide regarding the relationship between genetic structure within the species range and invasive properties. Both factors are related to dispersal ability, which, in turn, is dependent on the degree to which propagules can spread and to the capacity of a species to adapt to varying physical and biological environmental factors. A species may be unable to invade a certain area because it cannot reach it, or it may not do so because it cannot survive there. Its native populations will also be isolated from each other to the degree that propagules cannot overcome barriers within its native range. Such barriers can either hinder dispersal physically, or be environments that exceed the tolerance of propagules. Thus, native population structure and invasiveness should be related. Ultimately, the question is one regarding the factors that determine species ranges.

Mayr^6^ suggested that limits to range expansion are set by intraspecific gene flow. Populations at the range margin are unable to adapt to local conditions (and thus progressively invade new areas) because of the influx of genes from the species centre. Kirkpatrick and Barton^7^ constructed models that supported this proposal. Their reasoning would lead to the conclusion that species with high levels of genetic structure are more likely to expand their ranges. Existing data from invasive species, however, have indicated the opposite, *i.e.* that species with high rates of gene flow in their native range are more likely to expand to new areas. Bohning-Gaese, *et al*.^8^ found that in warblers of the genus *Sylvia* ability to disperse –as measured by capacity for long-distance flight– overwhelmingly explained the size of species ranges. In the marine realm, a meta-analysis by Gaither, *et al*.^9^ found that the best predictive variable of whether a species would become invasive is the inverse of genetic structure in its native range. Species with low pairwise (but not global) F_ST_ values between their native populations were more likely to be invasive. Thus, intraspecific phylogeography, of interest in any species, takes an additional dimension when the species is invasive, because it also addresses the basic biogeographical question of the factors that determine range expansion.

An invasive species with a wide native geographic range is the porcelain crab *Petrolisthes armatus* (Gibbes), a marine, crab-like anomuran decapod. This species has the largest geographical distribution of any neotropical porcellanid. In the Atlantic its range extends from central Florida and Bermuda to southern Brazil on the American coast, and from Senegal to Angola on the West African coast^10–12^. It is also present at Ascensión^13, 14^. In the eastern Pacific it ranges from the northern part of the Gulf of California to southern Perú, but it seems to be absent from the eastern Pacific offshore islands^12^. Until the early 1990s, the range of this species in the western Atlantic did not extent any farther north than Cape Canaveral^11, 12^. Rathbun^15^ listed a few specimens from Connecticut, but Chace^14^ expressed doubts regarding this report. Starting in the 1990s, *P. armatus* has expanded its populations to the shores of Georgia and South Carolina and has become abundant on rocky rubble, oyster reefs, and other intertidal habitats^16^, where it suppresses recruitment of other crabs and growth of small oysters^17^. Thus, the species is considered “invasive”^18–20^, although its recent spread is probably best classified as a range shift^18^, rather than an invasion.

The wide geographic distribution of *Petrolisthes armatus* is not due to an exceptionally long planktonic life. The planktonic phase, consisting of two zoea stages, in both eastern Pacific and western Atlantic populations ends in a settling megalopa in 12–19 days in the laboratory^21, 22^, a length of planktonic stage typical of other porcellanid species^23–28^. The wide distribution of the species and its potential to become invasive may be due to its wide habitat preference and physiological tolerance. The species occurs in the lower intertidal zone, under stones, in oyster and mussel beds, around mangrove roots, in corals, sponges and on dock pilings. It has also been dredged from rock, sand, and shell bottoms down to 18 m on the American coasts^12^ and down to 30 m in the eastern Atlantic^14^. *P. armatus* is by far the most abundant porcellanid in the rocky intertidal of the Panamanian Pacific coast, with densities up to 20 individuals per m^2^. It tolerates salinities as low as 10‰ inside the Panamá Canal Locks, where it is common^11^. According to a 16S rDNA phylogeny by Hiller, *et al*.^29^, *P. armatus* from both American coasts forms a sister clade to one comprised of *P. robsonae* and *P. zacae*, the former adapted to withstand great changes in salinity^12, 30^, and the later adapted to inhabit spaces formed by entangled roots of the mangrove *Rhizophora mangle* ^31^.

*Petrolisthes armatus* reproduces throughout the year both in eastern Pacific^12, 32^ and western Atlantic^33, 34^. Females of Pacific *P. armatus* release larvae synchronously near the times of nocturnal high tides^35^, probably as result of predation pressure by visual predators^36^. The larvae may use vertical migrations in order to be retained in estuaries^37–39^. Sexual maturity is reached in about a month after settlement.

Recent warming of coastal subtropical waters has facilitated a poleward range expansion of many organisms with tropical distribution^18^. It is not known whether the northward range shift of *P. armatus* along the coasts of Georgia and South Carolina was aided by warming waters, or whether larvae were not capable of spreading northward of Cape Canaveral until adults were transported by humans. Canning-Clode *et al*.^20^ studied the possible impact of cold water temperatures on the survivorship of *P. armatus*. In temperatures similar to the ones experienced by the southern and mid-Atlantic coast of the United States during abnormally cold-spells between January and March 2010, 39% of specimens survived.

Here we use DNA sequence data from the mitochondrial cytochrome oxidase I (COI) and 16S rDNA genes of *Petrolisthes armatus* collected from localities spanning a large part of its geographic range to address the following questions: (1) What does the intraspecific phylogeography of this species reveal about genetic connectivity of its populations? (2) Have there been barriers to gene flow within the species range? (3) How long ago were populations separated, and what has the rate of genetic exchange been after their separation? (4) Is the genetic structure of this invasive species consistent with the view that genetic structure is related to range expansions? (5) What is the origin of founders of newly established populations, and what have been the population genetic consequences of invasion of new areas?

## Results

### Phylogenetic Analyses

**Figure 1:**
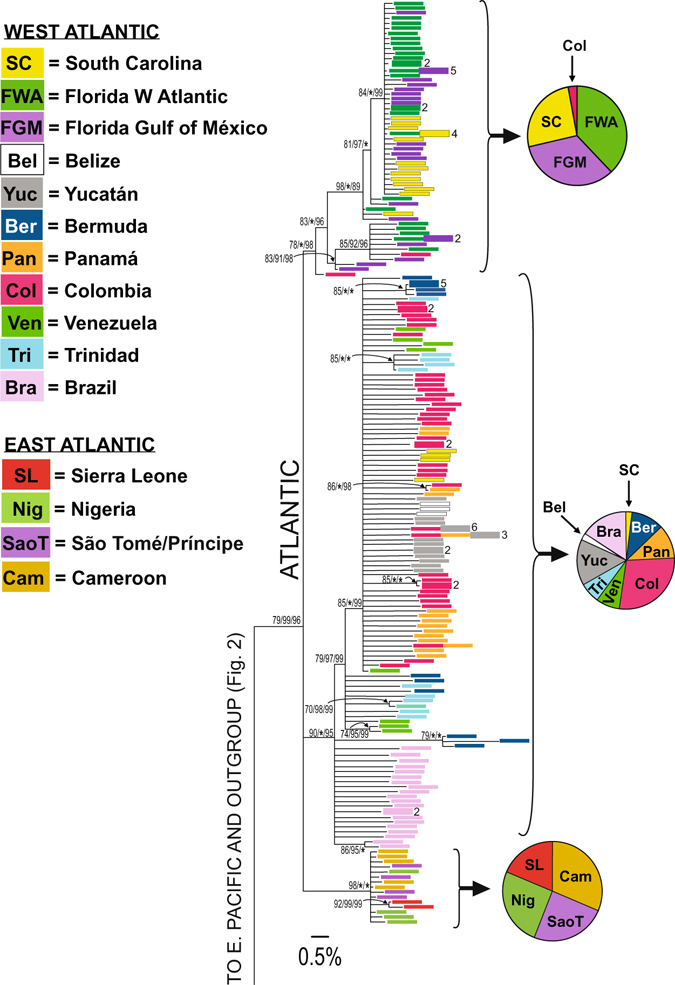
Gene genealogy of unique Atlantic haplotypes of concatenated 16S and two regions of COI of *Petrolisthes armatus*, showing a consensus tree from RAxML, MrBayes, and BEAST. Pacific haplotypes and outgroup are shown in Fig. 2. Colour bars indicate locality. Bars are followed by numbers when 2 or more identical sequences were found in each locality. Pie diagrams indicate the proportion of haplotypes from a particular locality encompassed in each clade. Values next to nodes indicate Maximum Likelihood/MrBayes/BEAST support in this order. *=100% Bayesian posterior probability support. Nodes with less than 70% support in RAxML or less than 85% in MrBayes and BEAST have been collapsed.

**Figure 2:**
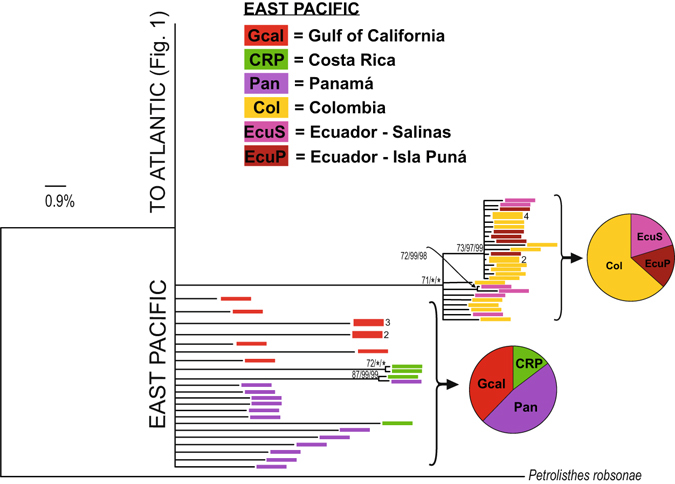
Continuation of phylogenetic tree shown in Fig. 1. Notation as in Fig. 1.

**Figure 3:**
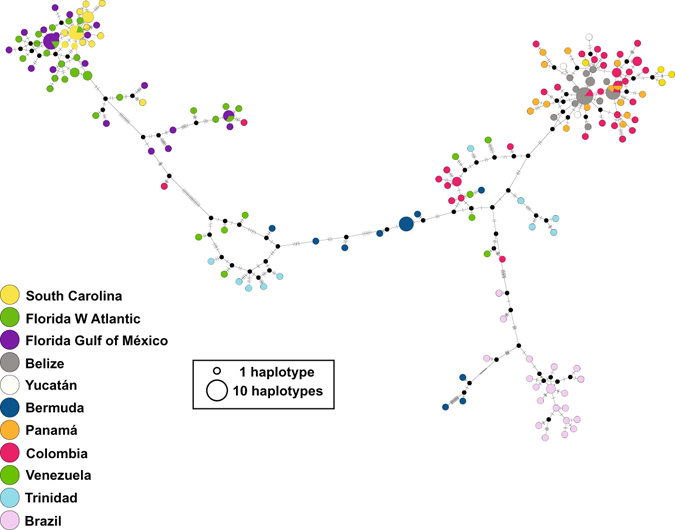
Median-Joining network of the West Atlantic clades of *Petrolisthes armatus*, based on concatenated haplotypes of 16S and two regions of COI. Colours indicate locality according to the legend, area of the circles illustrate the number of identical haplotypes, small black circles hypothetical ancestors or unsampled haplotypes, and hatch marks represent the number of mutations by which haplotypes differ.

Although intraspecific gene genealogies are said to be better represented by networks than phylogenetic trees because ancestral haplotypes may still exist in the sample^40^, the split in *Petrolisthes armatus* from the eastern Pacific and the Atlantic cannot be more recent than the completion of the central American Isthmus, and thus their common ancestor could not be younger than 3 MY. Our purpose of reconstructing a phylogeny was to identify and date major clades within the species. For this reason, we employed a conservative approach of collapsing every node that did not receive adequate support in both our Maximum Likelihood and the two Bayesian analyses. This mostly meant that we preserved all nodes supported by >70% of the bootstrap iterations in RAxML, because this program produced much lower clade support than either MrBayes or BEAST. The mtDNA phylogeny of *P. armatus* from concatenated 16S and COI sequences (Figs 1 and 2) revealed that Atlantic haplotypes are monophyletic. All three approaches provided high support for one clade on the seaboard of the United States (which we will name the “NW Atlantic” clade) and for a second clade with all haplotypes from Bermuda, and from all southern locations (the “SW Atlantic” clade). All three approaches also provided high support for a separate eastern Atlantic clade. There was, however, one discrepancy between the topology produced by BEAST, on the one hand, and by RAxML and MrBayes on the other. Whereas RAxML and MrBayes showed the Atlantic clade as a tritomy, BEAST showed the eastern Atlantic clade to be reciprocally monophyletic sister of the SW Atlantic clade with a posterior probability of 95%. According to estimates from BEAST, these two clades were separated 3.1 million years ago (MYA) [95% HPDs (Highest Posterior Density) 1.7 to 4.8 MYA], whereas, if assumed to be part of the tritomy, the estimated age of their most recent common ancestor would be the same as the age of the Atlantic clade, 3.5 MYA (95% HPDs 2.0 to 5.4 MYA). Given the 95% HPDs, these differences are not significant. The geographic separation of the NW and SW Atlantic clades is not absolute. Two haplotypes at Colombia belong to the NW Atlantic clade, and four haplotypes at South Carolina belong to the SW Atlantic clade (Figs 1 and 3). These phylogeographically “oddball” haplotypes by and large are not related to potential recolonization after the 2010 cold spell that is thought to have decimated the populations of *P. armatus* in the NW Atlantic. Only six haplotypes at South Carolina (Fig. 1), were collected in November 2012, whereas the rest were captured long before this time.

**Figure 4:**
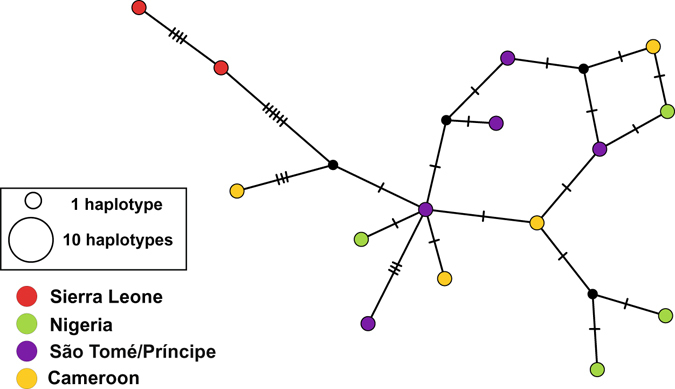
Median-Joining network of the East Atlantic clade of *Petrolisthes armatus*, based on concatenated haplotypes of 16S and two regions of COI. Notation as in Fig. 3.

Whereas the NW Atlantic clade shows little geographic partitioning, with haplotypes from the two sides of Florida and from South Carolina mixed, the SW Atlantic clade displays phylogeographic structure. Haplotypes from Brazil are closely related to each other (Fig. 3), and appear to be ancestral to haplotypes from all other localities in the southern clade (Caribbean and Bermuda), except for three haplotypes in Bermuda, which form a monophyletic entity, nested among the Brazilian haplotypes (Fig. 1). The separation between Brazilian and Caribbean subclades is dated by BEAST as having occurred 2.5 MYA (95% HPDs 1.4 to 3.9 MYA). The Caribbean clade contains a subclade composed of the majority of haplotypes from Colombia, Panamá, Yucatán and Belize, with a few haplotypes from Venezuela, South Carolina, Trinidad and Bermuda. This clade was estimated to be 1.7 MY old (95% HPDs 0.9 to 2.7 MYA), and is nested within a polytomy that contains haplotypes from the Southeast Caribbean and from Bermuda. The small sample of African populations shows little phylogeographic structure (Figs 1 and 4). Haplotypes from three locations in the Gulf of Guinea are closely related to each other, but more distantly related to two haplotypes from Sierra Leone.

**Figure 5:**
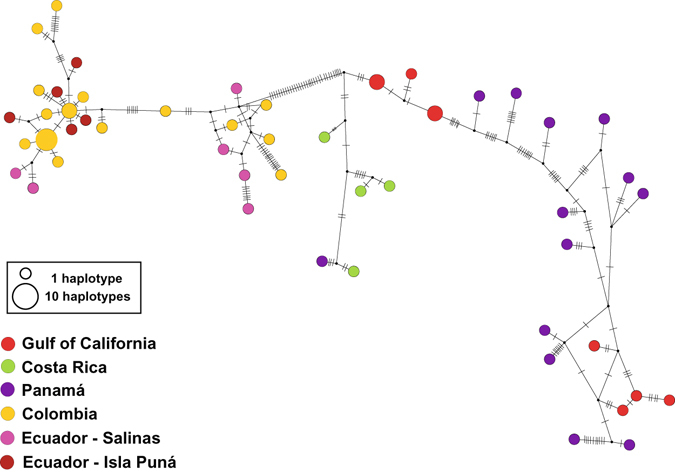
Median-Joining network of the East Pacific clade of *Petrolisthes armatus*, based on concatenated haplotypes of 16S and two regions of COI. Notation as in Fig. 3.

In the eastern Pacific (Fig. 2), haplotypes from Isla Puná and Salinas at the coast of Ecuador, and those from Colombia form a monophyletic unit with an estimated age of 1.8 MY (95% HPDs 0.7 to 3.5 MYA). However, the phylogeny of haplotypes from Panamá, Costa Rica and the Gulf of California is unresolved. Nevertheless the southern Pacific clade is separated from the rest of haplotypes by 33 mutations (Fig. 5). Given these phylogenetic divisions, analyses of population genetics were conducted within each of the three regions, the western Atlantic, the eastern Atlantic and the eastern Pacific.

### Population genetics

Hierarchical analysis of molecular variance (AMOVA) comparisons between clades (or geographically distant populations) illustrates the population genetic effect of population subdivision and few migrant haplotypes (Table 1). In the western Atlantic the gene genealogy suggested the existence of a barrier between Florida and Yucatán. Most of the variation (58%) was found on either side of the suspected barrier to genetic exchange, which is unsurprising given that these barriers were suggested by the phylogenetic analysis. Despite the existence of individuals of the northern clade in the South and vice-versa, and despite high F_ST_ values between populations in the same region, F_CT_ values between regions were high and significant. In the eastern Atlantic, the distance between Sierra Leone and the Gulf of Guinea may constitute a barrier to gene flow. Accordingly, 65% of the variation was between the population in Sierra Leone, on the one hand, and the populations in Cameroon, Nigeria and São Tomé, on the other; the F_CT_ value was high, but, because of small sample size in Sierra Leone, not significant. In the eastern Pacific 69% of the variation was explained by the separation of populations South and North of Panamá, but, once again, the F_CT_ value was not significant.

**Table 1:** Analysis of Molecular Variance (AMOVA), comparing populations on either side of a barrier suspected on the basis of phylogeny (see text) in each of three regions where *Petrolisthes armatus* occurs.

Region	Between regions		Between populations		Within populations	
	% Variation	F_CT_	% Variation	F_ST_	% Variation	F_SC_
Western Atlantic	57.60	**0.56**	14.83	**0.72**	27.58	**0.35**
Eastern Atlantic	65.00	0.65	0.77	**0.66**	34.23	0.02
Eastern Pacific	68.59	0.69	5.58	**0.74**	25.83	**0.18**

The comparison in the western Atlantic is between populations North and South of Florida (Florida populations were included in the North region), in the eastern Atlantic between Sierra Leone and three locations in the Gulf of Guinea, and in the eastern Pacific between North and South of Panamá (Panamá populations were included in the North region). Bold values indicate significant values on the basis of 10,000 reshufflings.

With the exception of immediately adjacent populations, pair-wise F_ST_ values between populations in the western Atlantic were large and highly significant (Table 2), which, under Wright’s^41^ island model would indicate a lack of gene flow over larger distances, assuming equilibrium between migration and genetic drift. Isolation by distance (IBD) analysis found a significant relationship between genetic dissimilarity and geographic distance, whether the two variables were measured in a linear (r = 0.506, p < 0.0001), or in a logarithmic (r = 0.541, p < 0.0004) scale. In contrast to the western Atlantic, genetic discontinuities between the populations in the eastern Pacific were mostly governed by the suggested barrier to gene flow between the coast of Colombia and the coast of Panamá. F_ST_ values between populations between these two areas were large and significant, whereas within each area –despite long geographic distances between Panamá and the Sea of Cortez– they were indicative of gene flow between distant populations. Correlations between genetic and geographic distance in the eastern Pacific were not significant in either a linear (r = 0.0496, p = 0.331) or a logarithmic (r = 0.0978, p = 0.263) scale. Very small and non-significant F_ST_ values between the Gulf of Guinea populations (Sierra Leone, with only two haplotypes was not included) indicated that *P. armatus* in this area is genetically uniform between Nigeria, Cameroon and São Tomé (Table 2). In the Isolation by Distance analysis the Mantel correlation coefficient was high (r = 0.9849), but because of the small number of populations and low sample size, not significant (p = 0.1644).

**Table 2:** F_ST_ values (below the diagonal) between populations in the same region, and times of separation of populations in years (above the diagonal) estimated from IMa2^111^, based on the concatenated fragment of 16S, COI-af, and COI-HL, except for comparisons involving Georgia (marked with an asterisk), which are based on COI-HL alone.

	Western Atlantic										
	Northern clade					Southern clade					
	Bermuda	Georgia*	S.Carolina	E Florida	W Florida	Yucatán	Panamá	Colombia	Venezuela	Trinidad	Brazil
Bermuda	—	NC	2,105,620	2,174,240	2,115,786	573,104	682,388	166,468	471,446	418,074	1,459,606
Georgia*	0.650***	—	25,462	31,875	1,713,262	NC	NC	NC	NC	NC	NC
S.Carolina	0.626***	−0.002NS	—	95,306	97,848	125,804	156,302	157,992	517,192	395,200	1,288,428
E Florida	0.697***	0.092**	0.101***	—	82,598	1,635,446	1,701,524	156,302	1,846,388	1,981,088	1,832,532
W Florida	0.673***	0.123***	0.085*	0.015NS	—	1,510,912	1,704,066	100,874	1,742,188	1,856,554	1,701,092
Yucatán	0.529***	0.598***	0.739***	0.803***	0.785***	—	110,554	163,318	634,100	456,196	184,934
Panamá	0.452***	0.560***	0.679***	0.758***	0.736***	0.045**	—	128,344	649,350	316,414	1,260,918
Colombia	0.313***	0.538***	0.624***	0.705***	0.686***	0.072**	0.0414NS	—	145,022	161,384	1,312,882
Venezuela	0.111*	0.668***	0.606**	0.687***	0.657***	0.556***	0.457***	0.271***	—	374,868	197,162
Trinidad	0.214***	0.653***	0.630***	0.708***	0.680***	0.540***	0.445***	0.296***	0.044NS	—	1,155,104
Brazil	0.586***	0.762***	0.735***	0.776***	0.757***	0.816***	0.760***	0.632***	0.590***	0.599***	—
**Eastern Atlantic**						**Eastern Pacific**					
	**Nigeria**	**Cameroon**	**São Tomé**				**Northern clade**		**Southern clade**		
Nigeria	—	85,140	151,320				Sea Cortez	Panamá	Colombia	Salinas	Isla Puná
Cameroon	0.001NS	—	115,910			Sea Cortez	—	>1,590,393	2,123,410	1,917,550	2,461,428
São Tomé	0.084NS	−0.022NS	—			Panamá	0.110NS	—	2,225,070	181,586	>2,655,172
						Colombia	0.746***	0.693***	—	143,594	87,682
						Salinas	0.684***	0.617***	0.178*	—	128,344
						Isla Puná	0.733***	0.663***	0.013NS	0.381*	—

***p < 0.001, **p < 0.01, *p < 0.05, NS: not significant. NC: IMa2 failed to converge. Separation of clades is based on the phylogeny shown in Figs 1 and 2.

F_ST_ statistics cannot distinguish between genetic similarity due to recent separation from similarity due to high gene flow. We employed a coalescent approach in IMa2 to date the time of the initial separation of populations and to estimate the magnitude and direction of subsequent gene flow. Based on the assumption that West Atlantic and eastern Pacific populations were separated for a minimum of 3 MY, and seeing that the average HKY^42^ distance (the model of DNA evolution used by IMa2) between their concatenated haplotypes is 4.04%, we have calibrated estimated times of divergence and migration between populations on the assumption that the 1718 bp fragment experiences a substitution rate per lineage of 7.5–15 mutations per MY. In comparisons between the population at Georgia and the rest of the samples (for which only a 499 bp fragment of COI-HL was available), we used the divergence in this DNA fragment between West Atlantic and Pacific populations of 5.02% to set the substitution rate prior for this fragment as 2–5 mutations per lineage per MY. Posterior probabilities of successful runs in IMa2 are supposed to rise to a peak at particular values of each parameter, then drop back down. In some pairwise comparisons, despite many chains, wide priors and long runs, posterior probabilities rose to a plateau and remained there, so that no unique value could be assigned to the relevant parameters. In the western Atlantic, times of initial separation estimated by IMa2 between populations (Table 2) ranged from tens of thousands to >2 million years. The most recent separations were between populations at South Carolina and Georgia, and between Georgia and East Florida, suggesting that the recently established northern populations may have been due to recent transfers. Obviously, given the recent introduction of *P. armatus* into Georgia and South Carolina, the estimates of separation of centuries to millions of years ago do not correspond to the actual time of transportation of individuals. These estimates of IMa2 are for the separation of the mixture of haplotypes transported into these localities relative to haplotypes in the rest of the species range. That populations at Georgia and South Carolina were the result of massive transfer, rather than having been started from a few individuals was also supported by their high genetic variability (Table 3). Both nucleotide and haplotype diversity in these populations were amongst the highest of all sampled populations with N > 10. Thus, the IMa2 results from the invaded localities reflect past history of the mixture of invaders. Estimates from the rest of the populations, however, are more likely to reflect population history.

**Table 3:** Nucleotide and haplotype diversity^107^ based on Tamura and Nei^109^ distances in sampled populations of *Petrolisthes armatus*. Concatenated data are composed of a partial sequence of 16S and COI-af and COI-HL. COI data are limited to COI-HL distances.

Location	N	Nucleotide diversity		Haplotype diversity	
		Concatenated	COI	Concatenated	COI
**West Atlantic**					
Bermuda	14	0.010	0.019	0.890	0.747
S.Carolina	22	0.009	0.007	0.961	0.697
Georgia	139	—	0.009	—	0.768
E Florida	26	0.008	0.011	0.994	0.572
W Florida	24	0.009	0.013	0.960	0.790
Yucatán	23	0.001	0.002	0.925	0.518
Panamá	15	0.003	0.004	1.000	0.905
Colombia	40	0.007	0.009	0.996	0.859
Venezuela	9	0.010	0.018	1.000	0.917
Trinidad	10	0.010	0.015	1.000	1.000
Brazil	21	0.004	0.001	0.995	0.516
**East Pacific**					
Sea of Cortez	10	0.011	0.043	0.911	0.844
Costa Rica	4	0.006	0.009	1.000	1.000
Panamá	13	0.013	0.031	1.000	0.949
Colombia	19	0.005	0.008	0.959	0.614
Salinas	6	0.006	0.006	1.000	0.800
Isla Puná	5	0.002	0.003	1.000	0.900
**East Atlantic**					
Sierra Leone	2	0.002	0.002	1.000	1.000
Cameroon	5	0.002	0.001	1.000	0.400
Nigeria	4	0.002	0.004	1.000	1.000
São Tomé	4	0.002	0.003	1.000	0.833

According to IMa2 estimates, the population of *Petrolisthes armatus* in Bermuda, the geographically most isolated locality in the western Atlantic, has been separated from all other localities 1.6 × 10^5^ to 2.1 × 10^6^ years ago (Table 2) and has received little gene flow since then, except from Trinidad and the coast of Colombia (Table 4). The four haplotypes of the SW Atlantic clade found in South Carolina (Figs 1 and 3) were interpreted by coalescence as having arrived from Bermuda, as well as Venezuela and Yucatán (Table 4). To the extent that the analysis of the shorter COI-HL fragment can be trusted, there has also been some gene flow from the founder haplotypes of Georgia into Bermuda (Table 4). The separation of the population in eastern and western Florida from populations that belong to the southern Caribbean clade has also been >10^5^ years ago (Table 2). The split between populations on the two coasts of Florida was estimated as much more recent. Gene flow between the two Florida populations has been relatively high, whereas gene flow into southern populations was not significantly different from 0 (Table 3). However, there has been asymmetrical gene flow from the Caribbean coast of Colombia, as also seen in the phylogenetic analysis (Figs 1 and 3). All estimated times of separation of populations within the SW Atlantic clade were in the order of 10^5^ years, except for those involving Brazil; the latter were more than a million years old with the exception of more recent isolation from Yucatán and Venezuela (Table 2). Most migration rates between Brazil and populations of the SW Atlantic clade were also not significantly different from 0 (Table 3). The contiguous Atlantic coasts of Panamá and Colombia registered both by F_ST_ values (Table 2) and by IMa2 estimates (Table 3) as containing a single, panmictic population. Populations at Yucatán and the Caribbean coast of Colombia appeared as exchanging a high number of propagules. Interestingly, the two NW Atlantic clade haplotypes found in Colombia (Figs 1 and 3) did not contribute to migration rates significantly different from 0 in IMa2.

**Table 4:** Rates of migration within each region estimated from the IMa2 algorithm^111^.

Western Atlantic												
		Northern clade						Southern Clade				
		Bermuda	Georgia*	S.Carolina	E Florida	W Florida	Yucatán	Panamá	Colombia	Venezuela	Trinidad	Brazil
		↓	↓	↓	↓	↓	↓	↓	↓	↓	↓	↓
Bermuda	←	—	1.133	0.000	0.000	0.000	0.000	0.000	**2.549**	NC	**8.746**	0.000
Georgia*	←	0.000	—	**19.740**	**15.740**	9.285	**4.436**	**4.451**	**4.460**	0.000	**0.327**	0.011
S.Carolina	←	**0.471**	1.249	—	0.000	0.150	**2.046**	0.000	0.000	**0.469**	0.000	0.000
E Florida	←	0.000	**2.249**	0.000	—	1.249	0.000	0.000	**1.373**	0.000	0.000	0.000
W Florida	←	0.000	NC	**2. 298**	NC	—	0.000	0.000	**2.249**	0.000	0.000	0.000
Yucatán	←	0.000	0.031	0.052	0.000	0.000	—	0.000	554.700	0.000	0.000	0.000
Panamá	←	0.000	0.000	0.000	0.000	0.000	0.000	—	1169.000	0.075	0.000	0.000
Colombia	←	0.000	0.000	0.000	0.000	0.000	NC	**1429.000**	—	0.000	0.000	0.000
Venezuela	←	0.000	0.000	0.000	0.000	0.000	0.000	0.000	0.000	—	0.000	0.000
Trinidad	←	0.000	0.200	0.000	0.000	0.000	0.000	0.000	0.000	11.153	—	0.000
Brazil	←	0.000	**0.318**	0.000	0.000	0.000	0.000	0.000	0.000	0.000	0.000	—
**Eastern Atlantic**						**Eastern Pacific**						
		**Nigeria**	**Cameroon**	**São Tomé**				**Northern clade**		**Southern clade**		
		↓	↓	↓				**Sea Cortez**	**Panamá**	**Colombia**	**Salinas**	**Isla Puná**
Nigeria	←	—	0.262	0.262				↓	↓	↓	↓	↓
Cameroon	←	0.262	—	0.000		Sea Cortez	←	—	NC	0.000	0.000	0.000
São Tomé	←	3.411	4.498	—		Panamá	←	0.000	—	0.000	0.000	0.000
						Colombia	←	0.000	0.000	—	0.000	7.496
						Salinas	←	0.000	0.000	0.000	—	**13.490**
						Isla Puná	←	0.000	0.000	2.499	**0.000**	—

Number of female propagules (2N_f_m) received by the population listed in first column from population listed in first row (in the direction of the arrows). NC: IMa2 failed to converge. Values are based on the concatenated fragment of 16S, COI-af and COI-HL, except for comparisons between Georgia (marked with asterisk) and the rest of the populations, which are based on COI-HL alone. Migration rates that were not significantly different from 0 are shown as “0.000”. Bold values indicate significantly asymmetrical gene flow. Clades as defined in Fig. 2.

In the eastern Pacific, estimated times of isolation between the population in the Sea of Cortez and any other population (including the one in Panamá) was >1.5 × 10^6^ years (Table 2). Despite their geographic proximity, but in agreement with their distant phylogenetic affinity, populations on the coast of Panamá and of Colombia were also estimated to have been initially separated >2 × 10^6^ years ago, longer than the isolation between populations at Panamá and the two localities at the coast of Ecuador (Salinas and Isla Puná). The most recent separation is between the Colombian and the Isla Puná populations. Rates of migration were generally not significantly different from 0, except for asymmetrical gene flow, from the population at Isla Puná to Salinas and from the coast of Colombia into Isla Puná (Table 3).

In contrast to the pattern seen in the western Atlantic, populations on the coasts of Nigeria, Cameroon and on the island of São Tomé contained haplotypes that mostly differ by only one mutation (Fig. 5), resulting in estimates of small F_ST_ values and recent separation (Table 2). Gene flow appeared to be along a North to South axis with a lower rate from São Tomé into Nigeria. Estimated migration in the opposite direction was not significantly different from 0.

## Discussion

Genetic structure of *Petrolisthes armatus* is characterized by deep phylogenetic divisions (*i.e.* clades) and large divergence between populations within the same clade. Clearly, this is a species with low ability to disperse, either within the western Atlantic or within the eastern Pacific, although our limited sample from the eastern Atlantic indicates higher genetic connectivity between the more closely spaced samples in this region. The degree of structuring within the West Atlantic and East Pacific is evident in the mtDNA phylogeny, in F_ST_ values and in the coalescent analysis. Yet a few errant haplotypes of one clade found in the region mainly inhabited by the other, along with the coalescence estimates of gene flow subsequent to initial separation, attest to infrequent long distance genetic exchange. What barriers and mechanisms of gene flow could account for the observed patterns?

Patterns of spread of genes between sedentary marine populations are obviously a function of the dispersal abilities of the species relative to potential barriers to planktonic transfer. The 12 to 19 day planktonic larva of *Petrolisthes armatus* ^21, 22^ is conducive to high population structure, but it is not so limiting, as to be the entire explanation for the deep divisions seen in this species. Other tropical species in the same regions, which in the laboratory complete their planktonic cycle in approximately the same period of time, such as the sea urchin *Echinometra lucunter* with a larval life of 19 days^43^, and the sponge *Cliona delitrix* with a larval life of about 10 days^44^, show much less genetic structure within the Caribbean. Only the sea anemone *Nematostella vectensis* ^45^, the gorgonian *Gorgonia ventalina* ^46^ and the barnacle *Chthamalus proteus* ^47^ show evidence of such high population differentiation between local populations. Shulman and Bermingham^48^ calculated that if larvae were transported as passive particles, it would take only 13 generations for genes of a species with a larva that stays in the plankton for two weeks to traverse through stepping stones the entire Caribbean following the main Caribbean Current, given average current vectors^49–52^. It is unlikely that *P. armatus* is lacking stepping stones, as, in addition to our sampled localities, it is also found in the Greater and the Lesser Antilles^10^. The lack of long-distance dispersal indicated by the high population structure, therefore, is more likely due to local retention of larvae, which appears to be a characteristic of this species^37, 38^. The tendency of larval retention, however, cannot be absolute in a species of such a wide range, so barriers to gene flow must also be present to account for the phylogeographic divisions.

An obvious such barrier is the Isthmus of Panamá, the timing of which we have used to date all other separations between populations. A multitude of lines of evidence has indicated that the final closure of the Isthmus, after a 12 MY process of narrowing water connections, occurred at approximately 3 MYA^53^. Some authors^54, 55^ have agreed that there were water connections until this time, but they presented their conclusions so as to imply that there may have been previous interruptions of gene flow between the eastern Pacific and the western Atlantic^56, 57^. Whether the water connections had narrowed at instances prior to 3 MYA bears little relevance to the phylogenetic reconstruction of *P. armatus*, because among five sister clades in the genus *Petrolisthes* with representatives on either side of the Isthmus of Panamá, eastern Pacific and Atlantic populations of *P. armatus* have diverged the least^29, 58, 59^, and are thus most likely to have been the most recently separated. This, however, does not necessarily mean that the trans-isthmian split was contemporaneous with the final isthmus closure, because it could have pre-dated it by any length of time. Our use of wide priors in the split between the Pacific and the Atlantic clades in the BEAST reconstruction has resulted in an estimated date of 3.5 MYA, which seems reasonable for a species with wide environmental tolerances, such as *P. armatus*. Based on this, we have estimated the split between western and eastern Atlantic clades at 3.1 MYA. The barrier that separated populations at these two regions was undoubtedly the long distance between American and African coasts. Scheltema^60^ estimated that, based on the speed of currents, larvae of marine organisms would require between 9 and 28 weeks to cross the contemporary tropical Atlantic. This distance barrier also accounts for distinct amphi-Atlantic clades in other porcellanids^29^, sea urchins^61, 62^ and fishes^63–66^. Given the short length of larval life of *Petrolisthes*, the puzzle is not how the separation happened, but rather how the original colonization was effected. The only explanation for the expansion of *P. armatus* to the coast of Africa is that this species can occasionally raft, possibly on mangrove roots, on which adults and juveniles are frequently found. We can only speculate about ocean circulation in the Pliocene, but modern-day currents between the coast of Brazil and the coast of Africa^67, 68^ are conducive to rafting transport; Ascensión may have been a stepping stone.

The barrier that could account for the presence of two clades in the Caribbean, one predominantly on the coast of Florida, the other from Yucatán to south Brazil, is less obvious. The population at Yucatán shows the lowest nucleotide diversity among all sampled localities (Table 3), suggesting that effective population size may be smaller. Richards, *et al*.^69^ found that a population of the brittle star *Ophiothrix suensoni* in the Gulf of Honduras was genetically isolated from populations in the rest of the Caribbean, which they attributed to larval entrapment arising from the Mesoamerican gyre^70, 71^. A similar explanation was offered by Jackson, *et al*.^72^ for the isolation of populations of the Nassau Grouper, *Epinephelus striatus* in this area. However, our sample from Yucatán was from the northern side of the peninsula and, given that in *Petrolisthes armatus* there is significant isolation by distance in the western Atlantic, the geographic separation of the two clades may be due to distance alone. Although larval entrapment in the Yucatán area could be the reason haplotypes in this area are so similar to each other, the F_ST_ values between the Yucatán population and populations on the coast of Colombia and Panamá are significant but relatively small (Table 2), so this population is not completely closed. There is no need to postulate a barrier between Yucatán and Florida, because the large genetic distance between them could simply be a reflection of more gradual differentiation between stepping stone populations off the Gulf of México shores, which were not sampled. What remains elusive is the original barrier that may have caused the differentiation of the NW and SW Atlantic clades. It is also difficult to explain why Bermuda was colonized only by the SW Atlantic clade. Presumably, the original colonization resulted from rafted individuals, carried by the Gulf Stream, which occasionally throws off cold rings that reach Bermuda^49^. The Gulf Stream, however, flows by the east coast of Florida, but we found no evidence of the existence of the NW Atlantic clade in Bermuda. Either the frequency of haplotypes of the northern clade in Bermuda is lower than ~0.07, so that it could not be detected with a sample of 14 individuals, or the colonization of Bermuda by southern haplotypes was a chance event that has not been repeated. The two haplotypes of the NW Atlantic clade in Colombia and the four haplotypes of the southern clade in South Carolina were interpreted by coalescence as being the result of migration, not of incomplete lineage sorting. To the extent that this reconstruction is correct, their ancestors must have arrived through the Antilles, because no such odd haplotypes were detected in any other sampled locality. The average vector of currents on the eastern side of the Antilles is from South to North^50^, so any movement in the opposite direction would likely be due to local tidal currents and wind. Populations of *P. armatus* on the eastern and western coasts of Florida appear to be connected by gene flow uncharacteristically high for this species. That organisms with higher capacity for dispersal than *P. armatus* show more pronounced genetic breaks on the two sides of the peninsula^73–77^ suggests human transport of this crab between these areas, similar to that responsible for the recent establishment of populations in Georgia and South Carolina. Haplotypes at the coast of Brazil in the MrBayes and BEAST phylogenetic reconstruction formed a monophyletic clade but they did not do so in RAxML. Nevertheless, the isolation of Brazilian populations is obvious in the high F_ST_ values and low estimates of migration in IMa2 (we consider the apparent migration from Georgia an artefact of the short COI-HL sequences). Isolation of Brazilian marine populations of other species from their conspecifics in the Caribbean is usually attributed to the fresh-water plume of the Orinoco and the Amazon^43, 62, 78, 79^; however, given the wide salinity tolerance of *P. armatus*, the isolation of its Brazilian populations may be due to the extensive mud habitat off the coast of Guyana and Surinam^80^.

In the eastern Pacific, the monophyletic clade composed of haplotypes at the coasts of Ecuador and Colombia, highly differentiated from those at Panamá and the Sea of Cortez, is unusual. No other organism sampled genetically to date displays this genetic break between populations at Colombia and Panamá^81^. There is ample rocky habitat between Ecuador and Panamá, and there is little in patterns of ocean circulation to suggest that larvae could not spread through stepping stones^82–84^. It will be interesting to find out whether other eastern Pacific species of *Petrolisthes* show a similar genetic discontinuity.

The newly established populations of *Petrolisthes armatus* in South Carolina and in Georgia are of particular interest, because they have earned the title of invasive species for this crustacean^1, 18–20^. Our data clearly show that these populations were initiated by the movement of individuals from Florida. Their haplotype and molecular diversity is as high as that of native populations, a frequent characteristic of invasive populations when they are established by a large number of propagules^1, 85–87^. Transfer of large numbers of the oyster *Crassostrea virginica* from the Gulf of México to the Chesapeake Bay during the early 1960s to replace oyster stocks infected with “MSX” disease has been associated with invasions by crustaceans inhabiting oyster beds from the Gulf to the Atlantic seaboard of the United States^88^; the same may have been true for introductions into Georgia and South Carolina. A large number of recently introduced founders, however, confounds the detection of evolutionary processes within the populations with the mark left by the genetic constitution of haplotypes that founded them. This is particularly true when there are multiple invasions from different sources^89, 90^. At the time that our samples were taken at Georgia and South Carolina, a maximum of 22 years had elapsed since *P. armatus* was noticed in these areas. Despite the obvious tendency of this species to form deep genetic divisions between its populations over time, ~250 generations have apparently not been enough for haplotypes to sort themselves to the degree that coalescence can correctly infer the history of isolation and migration. Comparisons of the Georgia population with those from the native range are further complicated by their being based on a short fragment of mtDNA, which is less likely to conform to the infinite sites mutation model assumed by the IMa2 algorithm. Population density of *P. armatus* in Georgia and South Carolina fluctuates widely^91^, as it also does in its native range^92, 93^, but the species appears well-established in areas occupied after its range shift^91^. *P. armatus* was present–though not as abundant–in South Carolina, even after the 2010 cold snap^20^, which suggests that larvae as well as adults are able to tolerate temperate temperatures. It is apparently too early to tell whether local adaptation in this marginal environment will permit the species to expand its range farther north.

## Conclusions

With sufficient variation, pronounced genetic structure is expected to allow local adaptation^6, 7^. Local adaptation is expected to promote range expansion^94, 95^. *Petrolisthes armatus*, according to our mtDNA data, is a species characterized by high structure, although this structure may need thousands or millions of generations before it becomes established so that local adaptation can ensue. The signature of natural obstacles to migration remains in the genetics of both the newly established and the old populations. Two major barriers are identified by the phylogeny, the separation of Atlantic and Pacific populations by the completion of the Isthmus of Panamá, and the almost contemporaneous separation of two clades on the opposite shores of the tropical Atlantic. The subsequent separation of the North and South clades in the western Atlantic and a similar break in the eastern Pacific do not lend themselves to obvious explanations as to their causes, but further support the conclusion that dispersal in *P. armatus* is limited. The recent range expansion from Florida to the northern Atlantic seaboard of the United States is most likely due to human transportation of oysters, but the ability of the species to survive in the new habitats shows that it possesses the necessary plasticity to tolerate new physical and biotic environments. Thus, the range limits of this species are more likely set by species characteristics related to low ability to disperse, rather than by narrow physiological tolerance. Given these characteristics, any subsequent spread of this invasive species is likely to be caused by human mediation.

## Material and Methods

### Collections

**Figure 6:**
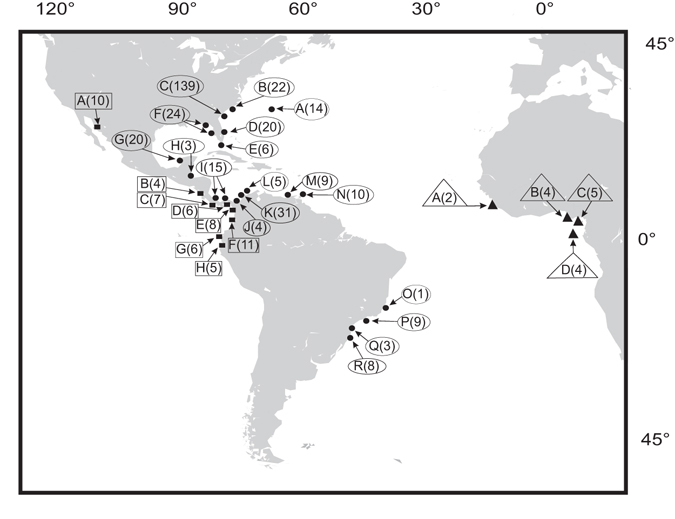
Collection localities of *Petrolisthes armatus*. Symbols and framed letters represent localities in each of the three oceans. Numbers in parentheses indicate sample size. WEST ATLANTIC: A: Bermuda; B: South Carolina: Conch Creek and Charleston; C: Georgia: Sapelo Island; D: Eastern coast of Florida: Fort Pierce; E: Florida Keys: Big Pine Key; F: Western coast of Florida: Tampa Bay and Dog Island; G: Yucatán Peninsula: Campeche; H: Belize: Tobacco Cay; I: Panamá: Bocas del Toro and entrance of the Panamá Canal; J: Colombia: Gulf of Urabá, Napú; K: Islas del Rosario and Gulf of Morrosquillo; L: Santa Marta; M: Venezuela: Isla Margarita; N: Trinidad; O: Brazil: Espírito Santo, Anchieta; P: São Paulo, São Sebastiao; Q: Paraná, Cabaraguara; R: Santa Catarina, São Francisco. EAST ATLANTIC: A: Sierra Leone: Freetown; B: Nigeria: Dowes Island; C: Cameroon: Limbe; D: São Tomé e Príncipe: Bom Bom Island. EAST PACIFIC: A: México: Sea of Cortez, Bahía Kino; B: Costa Rica: Bahía Salinas; C: Panamá: Chiriquí, Playa Hermosa; D: Ciudad de Panamá, Naos Island; E: Colombia: Chocó: Bahía Utría and Arusí; F: Valle del Cauca, Buenaventura and Bahía Málaga; G: Ecuador: Salinas; H: Isla Puná. Figure generated using the Map-It tool of the U.S. Geological Survey (https://woodshole.er.usgs.gov/mapit/). As a publication of the United States Government, it is in the public domain. No copyright exists or is necessary. Modifications to the basic map were made with CorelDraw® Home & Student X7 Ver. 17.1.0.572 (license No. DH17E224J7P25C62L2YZ77NPLP58) (http://www.coreldraw.com/en/all-products/).

We collected, or obtained from museums, 276 individuals of *Petrolisthes armatus* from both sides of the Atlantic and from the eastern Pacific (Fig. 6). All specimens from the Atlantic seaboard of the United States were collected before the extremely cold winter of 2010 (which is assumed to have caused high mortality of *Petrolisthes armatus* in this area^20^), except for 15 samples from Fort Pierce (collected in July 2010) and 11 samples from South Carolina (collected in November 2012). We additionally downloaded from GenBank 139 sequences of partial Cytochrome Oxidase I (COI) (Accession numbers FJ693377–693515) from Sapelo Island, Georgia, obtained by J.D. Robinson, E. Díaz-Ferguson, S. Pennings, T. Dale Bishop and J. Wares. These sequences were obtained from specimens collected in 2006–7 (J. Wares, per. com.).

### DNA extraction and sequencing

DNA was extracted from chelipeds or walking legs using the DNeasy^®^ Blood & Tissue Kit (Qiagen), following the manufacturer’s protocol for animal tissues. A 530 bp fragment of the ribosomal 16S rDNA was amplified using primers 16Sar-5′ (CGCCTGTTTATCAAAAACAT) and 16Sbr-3′ (CCGGTCTGAACTCAGATCACGT)^96^, and was trimmed to 508 bp in the alignment. A 640 bp of Cytochrome Oxidase I (COI) was amplified using primers COIf-5′ (CCTGCAGGAGGAGGAGAYCC) and COIa-3′ (AGTATAAGCGTCTGGGTAGTC)^96^, and was trimmed to 532 bp in the alignment. A second 680 bp COI fragment was amplified using primers LCO1490 (GGTCAACAAATCATAAAGATATTGG) and HCO2198 (TAAACTTCAGGGTGACCAAAAAATCA)^97^, and was trimmed to 678 bp in the alignment. We refer to the first COI fragment as COI-af and to the second as COI-HL. Double-stranded amplifications were performed in 25 ml volume reactions containing 5 µl of Taq buffer (5×), 2.5 µl of dNTP mix (8 mM), 1.2 µl of each primer (10 µM), 2.5 µl of MgCl_2_ (25 mM), 0.2 µl of GoTaq® Flexi DNA *Taq* Polymerase (Promega), 1 µl of DNA template, and 11.4 µl of ddH_2_O. Thermal cycling conditions consisted of an initial denaturation step at 96 °C for 3 min, followed by 30 cycles of 95 °C for 1 min, 50 °C for 1 min, and 72 °C for 1 min. An extension step at 72 °C for 5 min followed the last cycle. Amplifications that resulted in unique PCR products were cleaned using the ExoSap-IT^®^ kit (USB Corporation) following the manufacturer’s protocol. Samples that amplified multiple products were purified by cutting the band of the appropriate molecular weight out of a 2% low-melt agarose gel after electrophoresis in 1× TAE buffer. PCR products were recovered by incubating the sample at 70 °C for 10 min, and then at 45 °C for 60 min after adding 1 µl of GELase™ (Epicentre Biotechnologies). Clean PCR products were cycle-sequenced in both directions using the BigDye^®^ Terminator v. 3.1 Cycle Sequencing Kit, and electrophoresed in an Applied Biosystems® 3130 Genetic Analyzer. DNA from one individual of *Petrolisthes robsonae* was also sequenced to be used as outgroup in the phylogenetic analyses. Sequences were aligned by eye using BioEdit^98^. Multiple attempts to identify an informative nuclear sequence failed.

### Phylogenetic analyses

The Akaike Information Criterion [AIC; ref. 99], implemented in the program jModeltest2^100^, was used to select the model of nucleotide substitution that best fit each DNA region. These models were TIM3+I (I = 0.92) for 16S, GTR+I+G (I = 0.74, α = 1.29) for COI-af, and TIM+I+G (I = 0.18, α = 0.14) for COI-HL. The three fragments of each individual were concatenated and subjected to partitioned phylogenetic analysis after removal of redundant haplotypes, with the appropriate model applied to each partition.

Maximum Likelihood (ML) phylogenetic reconstruction was conducted in RAxML v. 8.2.6^101^, applying a GTR model with different values of α for each partition, as indicated by jModelTest2, and using the options for rapid bootstraps and automatic halting. Support values for the nodes were estimated from 456 bootstraps. Bayesian reconstruction was conducted in two Bayesian algorithms, MrBayes v.3.2.2^102^ and BEAST v. 1.8.2^103^, using as priors the models indicated by jModeltest2, but letting the programs estimate the parameters. Mr. Bayes was run in 4 chains and for 6 × 10^7^ steps, which allowed the average standard deviation of split frequencies to fall below 0.01, and the potential scale reduction factor to be equal to 1.00. Convergence was also determined in multiple runs, which produced the same topology. Node credibility values were determined by sampling every 600^th^ tree after discarding 25,000 trees as a burnin. BEAST results came from eight separate runs of different numbers of steps, combined in Logcombiner v. 1.8.2 taking every 10^th^ tree and a burnin of 10% per run, for a total of 26,085 trees. Tracer v. 1.6 verified that Effective Sample Size (ESS) of all estimates of the combined runs exceeded 200.

In addition to reconstructing the phylogeny, BEAST was used to estimate date of divergence between major clades. For this purpose, the separation between Atlantic and Pacific haplotypes was given an offset of 3 million years (MY) in a Lognormal Uncorrelated Relaxed clock. This is the generally accepted date of the completion of the Central American Isthmus^53, 59, 104^. However, as there are claims that only “narrow, shallow, and transient channels” connected the Caribbean with the Tropical Pacific as of 13 MYA (million years ago)^54^, the priors for this calibration point were set as uniform, ranging from 0.2 to 10^100^ MY. RAxML and BEAST analyses were run on the CIPRES Science Gateway^105^. A median-Joining network^106^ of haplotypes in the same geographic region was constructed in PopArt v.1 (http://popart.otago.ac.nz) to visualize relationships between haplotypes and geography within subclades in which the ancestral sequences may still be present in the populations^40^.

### Population genetic analyses

For the purposes of population genetic analyses, 4 samples from the Pacific shore of Costa Rica were pooled with those from the adjacent coast of Panamá, and 3 samples from Belize were pooled with those from Yucatán. All analyses were based on the full concatenated sequences of 16S, COI-af and COI-HL except for those involving the invasive Georgia population, for which only sequences from COI-HL were available. Haplotype and molecular diversity^107^ and sequence-based pairwise F_ST_ (Φ_ST_) values were calculated in Arlequin 3.5.1.2^108^ using Tamura and Nei^109^ distances. Analysis of Molecular Variance (AMOVA)^110^ was also carried out with this program, with significance determined in 10,000 reshufflings of haplotypes between populations. For a coalescent analysis, intended to determine times of separation of populations and subsequent direction of gene flow, we used IMa2 v. 2.0^111^. In the MCMC mode of this program we used geometric heating with 60 chains and a burnin of 5 hours. The runs were continued until an ESS of >200 was obtained for each parameter. L mode, intended to determine whether gene flow was significantly different from 0 and significantly different in each direction, was run for 3 × 10^7^ steps. The uniform prior for mutation rate per lineage was estimated from assuming that divergence between Atlantic and Pacific populations of *P. armatus* reflects separation of 3 MY. The prior of the substitution rate was given a range equal to the mean and distributed symmetrically on either side of the mean. Comparisons between populations were made in pairwise fashion in separate runs. For Isolation by Distance analysis, Mantel^112^ correlations between Φ_ST_ and geographic distance by sea we employed IBDWS^113^ with 10^4^ iterations.

### Data Availability

Datasets generated during the current study are available in GenBank under accession numbers KY856997–857273 (for 16S rDNA) and KY857274–857550 (for COI) and in Dryad (https://doi:10.5061/dryad.pm1r2) (alignments).
